# Automated segmentation of liver and hepatic vessels on portal venous phase computed tomography images using a deep learning algorithm

**DOI:** 10.1002/acm2.14397

**Published:** 2024-05-21

**Authors:** Shengwei Li, Xiao‐Guang Li, Fanyu Zhou, Yumeng Zhang, Zhixin Bie, Lin Cheng, Jinzhao Peng, Bin Li

**Affiliations:** ^1^ Minimally Invasive Tumor Therapy Center Beijing Hospital Peking Union Medical College Beijing China

**Keywords:** computed tomography, deep learning, liver, segmentation

## Abstract

**Background:**

CT‐image segmentation for liver and hepatic vessels can facilitate liver surgical planning. However, time‐consuming process and inter‐observer variations of manual segmentation have limited wider application in clinical practice.

**Purpose:**

Our study aimed to propose an automated deep learning (DL) segmentation algorithm for liver and hepatic vessels on portal venous phase CT images.

**Methods:**

This retrospective study was performed to develop a coarse‐to‐fine DL‐based algorithm that was trained, validated, and tested using private 413, 52, and 50 portal venous phase CT images, respectively. Additionally, the performance of the DL algorithm was extensively evaluated and compared with manual segmentation using an independent clinical dataset of preoperative contrast‐enhanced CT images from 44 patients with hepatic focal lesions. The accuracy of DL‐based segmentation was quantitatively evaluated using the Dice Similarity Coefficient (DSC) and complementary metrics [Normalized Surface Dice (NSD) and Hausdorff distance_95 (HD95) for liver segmentation, Recall and Precision for hepatic vessel segmentation]. The processing time for DL and manual segmentation was also compared.

**Results:**

Our DL algorithm achieved accurate liver segmentation with DSC of 0.98, NSD of 0.92, and HD95 of 1.52 mm. DL‐segmentation of hepatic veins, portal veins, and inferior vena cava attained DSC of 0.86, 0.89, and 0.94, respectively. Compared with the manual approach, the DL algorithm significantly outperformed with better segmentation results for both liver and hepatic vessels, with higher accuracy of liver and hepatic vessel segmentation (all *p* < 0.001) in independent 44 clinical data. In addition, the DL method significantly reduced the manual processing time of clinical postprocessing (*p* < 0.001).

**Conclusions:**

The proposed DL algorithm potentially enabled accurate and rapid segmentation for liver and hepatic vessels using portal venous phase contrast CT images.

## 1 INTRODUCTION

Accurate segmentation from medical images is a fundamental prerequisite for surgical planning.^1^ Multiphase computed tomography (CT) remains the preferred imaging modality for hepatic lesions.^2^, ^3^ Due to the intricate hepatic anatomy, even experienced surgeons can be blinded to some critical structures, potentially affecting surgical decision‐making.^4^ Accurate information acquisition on the liver and hepatic vessels from CT images greatly facilitates preoperative planning of hepatectomy, liver transplantation, and minimally invasive therapies.^5^, ^6^, ^7^ Prior liver segmentation mainly concentrates on contour structures and volumetric measurements, while the segmentation for both internal anatomy and vascular architecture remains limited.^8^, ^9^, ^10^

Conventional image segmentation is manually performed by radiologists, which is time‐consuming and subjective to substantial inter‐observer variations.^11^ Since manual approaches cannot satisfy surgical planning within a short time,^12^ automated segmentation has attracted increasing interest. Affected by anatomical variations and complexity, the accuracy of automated liver and hepatic vessel image segmentation hardly reaches clinical satisfaction without operator interactions.^7^, ^13^, ^14^, ^15^ Recently, deep learning (DL) development has enabled convolutional neural networks (CNNs) to be increasingly applied to automated image segmentation.^16^, ^17^, ^18^, ^19^ These algorithms automatically segment medical images in an end‐to‐end manner and progressively improve parameters to optimize final segmentation, generally achieving better performance. Several CNN frameworks have been proposed for medical image segmentation, including fully convolutional networks (FCNs),^20^ DeepLab,^21^ dense convolutional networks (DenseNets),^22^ residual networks (ResNets),^23^ generative adversarial networks (GANs),^24^ and U‐shaped networks (U‐Nets).^25^ Among these, U‐Net architecture with promising results has widely attracted popularity. For example, the Attention‐Based Residual U‐Net proposed by Wang et al.^26^ achieved automatic and accurate liver segmentation on LiTS17 and SLiver07 datasets. Kitrungrotsakul et al.^27^ also introduced a deep CNN for accurate hepatic vessel segmentation. Due to complicated and changeable imaging representations, the accuracy of DL algorithms relies upon the quantity and variety of training data;^28^ however, these networks were developed based on limited training samples from public datasets and evaluated using the reference datasets with incomplete annotations. Particularly, annotation quality of datasets highly affects the accuracy of DL algorithms, and considering incomplete annotations as the benchmark for training and evaluating may cause segmentation bias.

Hence, our study aimed to propose a DL algorithm for accurate and rapid liver and hepatic vessel segmentation based on sufficient CT data with high‐quality annotations. Furthermore, the DL algorithm was clinically evaluated using preoperative CT data from patients with focal lesions.

## 2 METHODS AND MATERIALS

This study was approved by the institutional research ethical committee and the written informed consent was waived due to the retrospective nature of this study. We present our article in accordance with the STROBE reporting checklist.

### 2.1 Datasets

#### 2.1.1 The development and test datasets

Our study was retrospectively performed based on two clinical datasets (Development dataset and Test dataset). Development dataset was composed of 515 multiphase contrast‐enhanced abdominal CT scans in XXX Hospital from January 2021 to December 2022. Test dataset was established from preoperative portal venous phase CT images from 44 patients with hepatic focal lesions undergoing CT‐guided percutaneous liver parenchymal biopsy in XXX Hospital from January 2023 to June 2023. The details of two datasets are briefly summarized in Table 1.

**TABLE 1:** Characteristics of the private dataset and independent test dataset.

	Datasets	
Characteristics	Development dataset	Independent test dataset
No. of patients	515	44
Age (year)*	55.0 ± 13.7	56.8 ± 12.9
Gender (Male/Female)	288/227 (56.0%/44.0%)	25/19 (56.8%/43.2%)
Focal hepatic lesions		
No	424 (82.3%)	0
Hepatic cyst	26 (5.1%)	2 (4.5%)
Benign tumor	10 (1.9%)	2 (4.5%)
Hepatocellular carcinoma	49 (9.5%)	32 (72.7%)
Metastasis	4(0.7%)	7 (15.9%)
Other malignancy	2 (0.4%)	1 (2.3%)
Spatial resolution		
Voxel spacing (mm)*		
x‐axis	0.72 ± 0.05	0.72 ± 0.05
y‐axis	0.72 ± 0.05	0.72 ± 0.05
z‐axis	0.69 ± 0.02	0.69 ± 0.02

*Note*: Data are expressed as the number of cases while the data in parentheses are percentages. *Data are expressed as mean ± standard deviation.

The included CT scans were strictly selected through the inclusion and exclusion criteria. Inclusion criteria: (1) DICOM format data with integrity; (2) ≤2 mm slice thickness and matrix size ≥512 × 512; (3) Multiphase or only portal‐venous phase contrast enhanced CT images covering the entire liver. Exclusion criteria: (1) Incomplete CT series; (2) Inadequate scanning phases or ranges. Multiphase contrast‐enhanced CT scans were acquired by a GE Discovery 16 Slice CT scanner (GE Health care, Boston, USA). The portal venous phase images were stored in DICOM format and presented with slice thickness of 1.25 mm. All the data were completely anonymized and no patient‐specific information was extracted or could be retracted.

In the development dataset, 413, 52, and 50 CT scans were applied to the training, validation and testing of the DL algorithm, respectively. Additionally, its robustness and generalization were independently evaluated based on 44 CT scans in the test dataset.

#### 2.1.2 The annotation protocol and ground truth

To ensure the consistency of segmentation results, the radiologists involved with this project went through a training procedure and annotated several cases together before formal annotation. To obtain the reliable ground truth, each CT series was manually annotated by one of three experienced radiologists (Dr. S. L., Dr. J. P., and Dr. B. L., with 8‐, 10‐, and 12‐year experience in abdominal radiology, respectively) under the supervision of one expert radiologist (Dr. Z.B., with 15‐year experience in abdominal radiology). Firstly, all the images were randomly divided into three groups and evenly distributed to each radiologist. The preliminary masks marking the liver and hepatic vessels were generated and prelabeled using ITK‐SNAP 3.6 software. To reconfirm the accuracy, the preliminary masks were independently double‐checked and refined by one expert radiologist (Prof. X.L., with more than 20 years of experience in abdominal radiology). Any inaccuracies were adjusted and corrected by the expert radiologist, after which the final segmentation masks were accomplished as the ground‐truth reference. This annotation protocol was globally applied to the manual ground‐truth generations in our study.

### 2.2 Data pre‐processing and augmentation

Data pre‐processing plays a crucial role in medical image segmentation tasks. The pre‐processing of the CT images mainly included the following steps. Firstly, the images were reorientated to the LPI(Left‐Posterior‐Inferior)direction in patient coordinate system. Then, CT images were resampled to a fixed size with a coarse input of 512 × 512 and a fine output of 256 × 256 to reduce the computational loads. The Hounsfield values were windowed with range from −200 to 350 to enhance the contrast and remove the irrelevant tissues from CT images. In addition, a z‐score normalization was applied based on the mean and standard deviation of the intensity values.

To alleviate data overfitting, data augmentation methods were used during training. Specifically, we augmented the training data by horizontal flipping, rotation with a maximum angle of 25°, shifting by horizontally and vertically translating the images (a maximum of 20 pixels), random occlusion of erasing a rectangle imaging region with a random value and adding Gaussian noise to the data. The rotation and shift were used to increase the adaptability to different body positions in the surgical scanning. The random occlusion aimed to avoid the metal implant artifacts and Gaussian noise was added to improve the robustness.

### 2.3 The proposed DL algorithm development

Our developed DL algorithm performed the training, validation and testing based on 413, 52 and 50 CT scans from the Development dataset, respectively.

**FIGURE 1:**
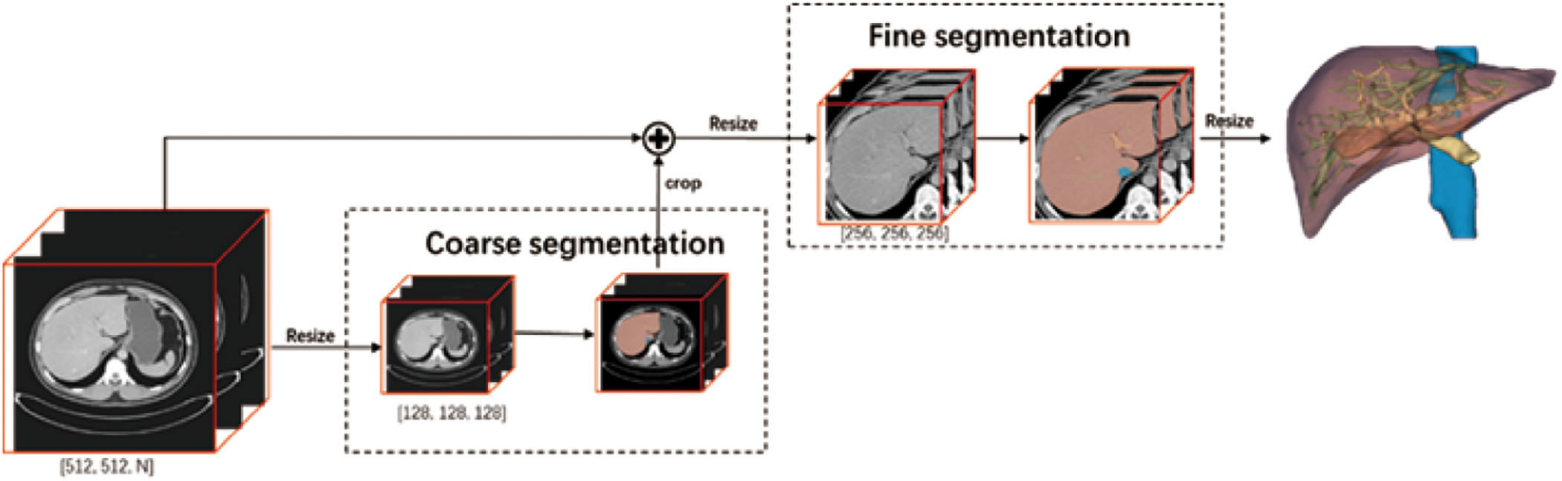
A schematic diagram of a whole‐volume‐based coarse‐to‐fine segmentation framework.

Our DL algorithm using a whole‐volume‐based coarse‐to‐fine framework^29^ mainly composed of coarse and fine segmentation (Figure 1). Briefly, the coarse segmentation process preliminarily extracted the general features based on the whole‐volume CT images, such as the position and contours. After that, the results were further refined in details during the fine process. Theoretically, this coarse‐to‐fine segmentation pattern was highly efficiently and adaptable to huge anatomical variations in hepatic regions.

**FIGURE 2:**
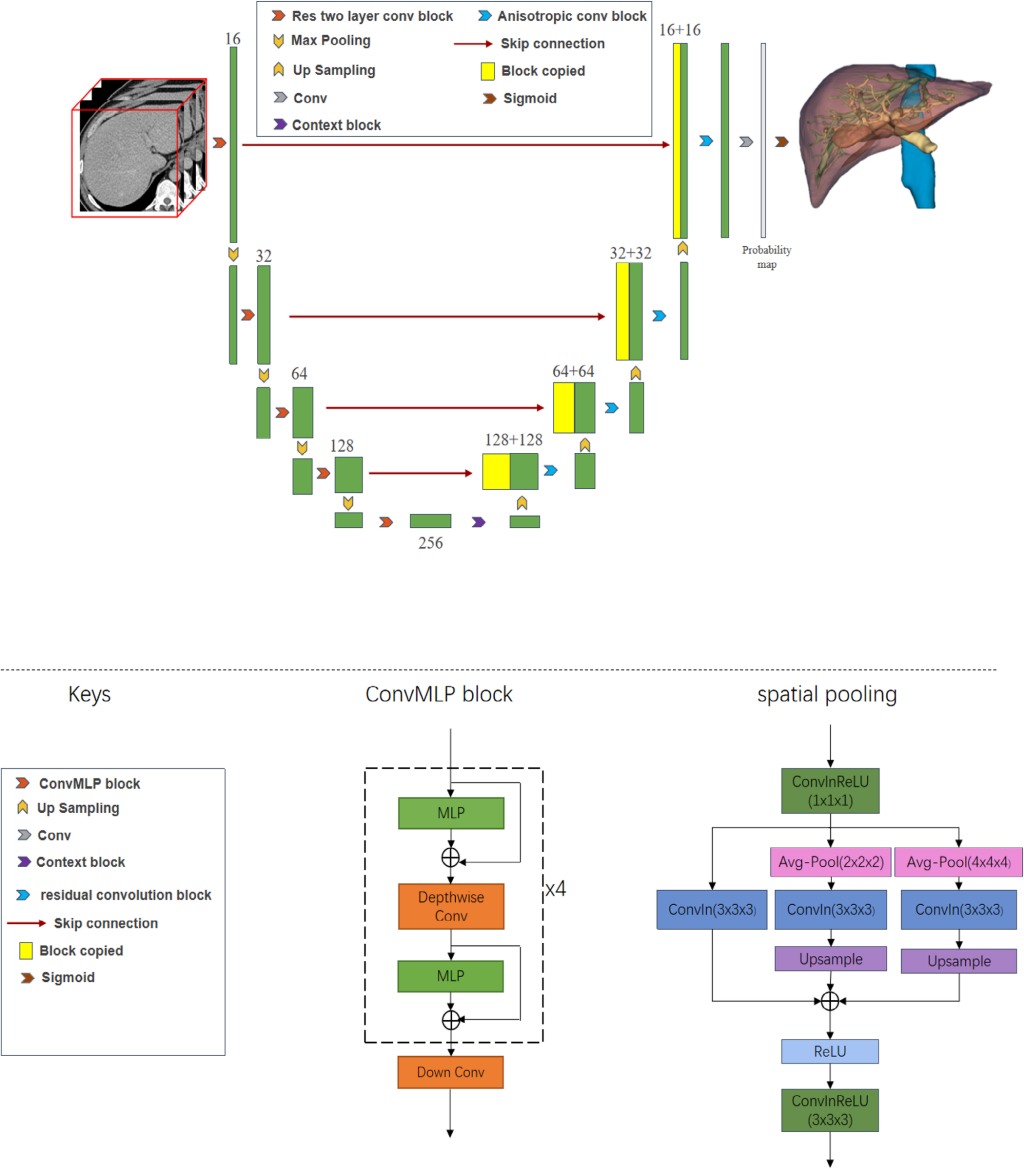
The architecture of the coarse‐to‐fine deep learning segmentation U‐net framework.

The proposed U‐Net deep learning algorithm consists of three major parts: the feature encoder module, the context extractor module, and the feature decoder module (Figure 2). The encoder module is composed of a consecutive multi‐layer perceptron (ConvMLP) block,^30^ and the decoder module with one residual convolution block. The details of the DL algorithm are described in Supplementary Materials 1.

### 2.4 Validation in preoperative clinical data

The performance of DL‐based segmentation was extensively evaluated and compared with manual segmentation using preoperative 44 CT scans with focal lesions from real clinical scenarios. The DL‐based automated segmentations were accomplished using the DL algorithm, while the manual segmentations were implemented by the radiologists (Dr. S. L., Dr. J. P., Dr. B. L.) in clinical practice using the Volume Viewer in Medical Image Processing Software (GE Medical Systems SCS, GE Health care, Boston, USA), which is the widely used software in medical image‐postprocessing. All 44 CT series was annotated in accordance with the standard annotation protocol (Section 2.1.2) to generate the ground truths and all manual segmentations were independently performed blinded to the DL‐derived results. Finally, the evaluation metrics and image processing time were quantitatively compared. The manual processing time per case was recorded from the first ROI setting until the final revision, while the DL processing time per case was from the initial input of images to the final output. Additionally, our proposed DL method was compared to the CNN^16^ and Unet^26^, ^27^ networks based on 44 CT series in the Test dataset.

### 2.5 Inter‐observer and inter‐method agreement

To evaluate inter‐observer variability among the radiologists in manual segmentation, the manual segmentation was performed by the three radiologists Reader 1 (Dr. S. L.), Reader 2 (Dr. J. P.), and Reader 3 (Dr. B. L.) on a randomly selected subgroup of 40 CT series, each. All manual segmentations were performed blinded to the results from other radiologists. The inter‐observer agreements of the DSC values were estimated using the intraclass correlation coefficients (ICCs).

To assess the differences between the two methods, the DL‐derived DSC values were compared to the averaged DSC of manual segmentations from three radiologists (Reader 1, 2, 3). Another subgroup of 44 CT series was randomly selected for the inter‐method analysis using Bland–Altman plots. All manual segmentations were performed blinded to the DL‐derived results.

### 2.6 Evaluation metrics

The accuracy of DL‐based segmentation was quantitatively evaluated using the Dice Similarity Coefficient (DSC), Normalized Surface Dice (NSD) and Hausdorff distance_95 (HD95).

Different from the liver parenchymal, the hepatic vessels are generally tube‐structured branches with the highly skewed proportion of vessels and background. Recall and Precision are more frequently adopted in vessel segmentation assessment by excluding true‐negative cases (pixels belonging to background in accordance with GS) from comparisons.^31^ Besides DSC, Recall and Precision are used to evaluate the segmentation performance of hepatic veins, portal veins, and inferior vena cava in our study. The definition of evaluation metrics is shown in Supplementary Materials 2.

### 2.7 Statistical analysis

Data are represented as mean ± standard deviation. The inter‐reader and inter‐method agreements were assessed using the intraclass correlation coefficient (ICCs) with corresponding 95 % confidence intervals (CI) and the Bland–Altman 95% limits of agreement (LOA), respectively. Comparison of DSC was calculated via the paired samples *t*‐test, while the NSD, HD95, Recall, Precision and processing time comparisons were computed using paired samples Wilcoxon signed rank test. *p* value < 0.001 shows statistically significant difference. Statistical analysis was performed using SPSS version 21.0 (IBM Corp., Armonk, NY, USA).

## 3 RESULTS

### 3.1 Segmentation performance of the proposed network

After the initial training procedure using 413 data, the accuracy of DL‐based liver and hepatic vessel segmentation was quantitively evaluated using 52 (validation) and 50 (test) CT scans, respectively.

**FIGURE 3:**
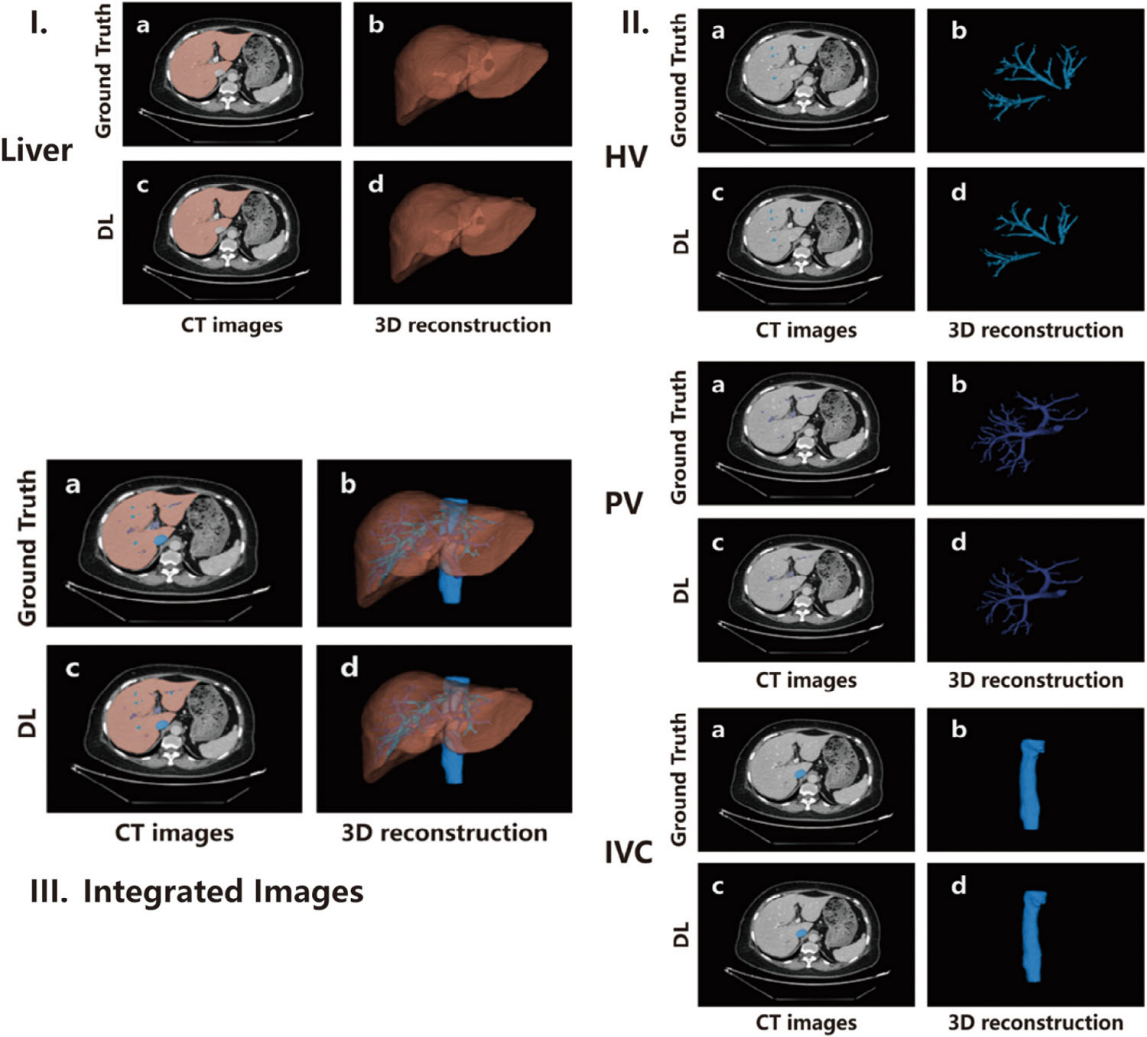
Representative segmentation and 3D reconstruction based on the developed DL algorithm. (I) DL‐based liver segmentation and 3D reconstruction, (II) DL‐based segmentation and 3D reconstruction of the hepatic vessels (hepatic vein, portal vein, and inferior vena cava), (III) Integrated illustration of the DL segmentation. In each imaging group, (a) Image of the ground truth segmentation, (b) 3D reconstruction based on the ground truth segmentation, (c) Image of the DL segmentation, (d) 3D reconstruction based on the DL segmentation. DL, deep learning; HV, hepatic vein; IVC, inferior vena cava; PV, portal vein.

The DL‐based liver segmentation achieved the highest accuracy with DSC of 0.98 ± 0.01, NSD of 0.92 ± 0.04, and HD95 of 1.52 ± 0.89 mm in 50 testing cases, while DSC of 0.98 ± 0.01, NSD of 0.89 ± 0.06, and HD95 of 1.88 ± 0.81 mm in 52 validation cases. The quantitative evaluation of the DL algorithm was demonstrated in Table 2. Compared with the ground‐truth, the representative DL‐based segmentation results were illustrated in Figure 3.

**TABLE 2:** Quantitative evaluation of the DL algorithm for liver segmentation.

Performance	Dataset	*N*	DSC	NSD	HD95 (mm)
Liver segmentation	Validation	52	0.98 ± 0.01	0.89 ± 0.06	1.88 ± 0.81
	Test	50	0.98 ± 0.01	0.92 ± 0.04	1.52 ± 0.89

*Note*: Data are represented as mean ± standard deviation.

Abbreviations: HD95, Hausdorff distance_95; DSC, dice similarity coefficient; NSD, normalized surface dice.

As for hepatic vessel segmentation, our DL algorithm successfully attained accurate results in 50 testing data with DSC of 0.89 ± 0.02, 0.86 ± 0.03, and 0.94 ± 0.02, Recall of 0.87 ± 0.04, 0.84 ± 0.06 and 0.95 ± 0.03, Precision of 0.92 ± 0.03, 0.89 ± 0.03, and 0.93 ± 0.03 for portal veins, hepatic veins, and inferior vena cava, respectively. The assessments of DL‐based segmentation for these hepatic vessels were quantitatively shown in Table 3. The representative segmentations and 3D vascular reconstructions of the hepatic veins, portal veins, and inferior vena cava were illustrated (Figure 3). Moreover, the integrated illustration of the DL‐based segmentations for the liver and hepatic vessel is shown in Figure 3.

**TABLE 3:** Quantitative evaluation of the DL algorithm for hepatic vessel segmentation.

Performance	Dataset	*N*	DSC	Recall	Precision
Hepatic veins	Validation	52	0.83 ± 0.11	0.81 ± 0.13	0.87 ± 0.11
	Test	50	0.86 ± 0.03	0.84 ± 0.06	0.89 ± 0.03
Portal veins	Validation	52	0.89 ± 0.03	0.87 ± 0.04	0.91 ± 0.05
	Test	50	0.89 ± 0.02	0.87 ± 0.04	0.92 ± 0.03
Inferior vena cava	Validation	52	0.92 ± 0.04	0.93 ± 0.05	0.91 ± 0.06
	Test	50	0.94 ± 0.02	0.95 ± 0.03	0.93 ± 0.03

*Note*: Data are represented as mean ± standard deviation. DSC = Dice similarity coefficient.

### 3.2 Validation in preoperative clinical data

**FIGURE 4:**
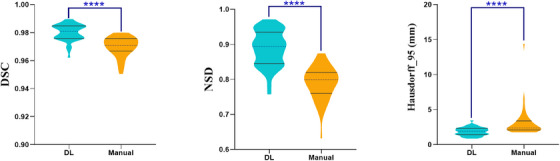
Violin plots of the comparisons between the DL algorithm and manual method on the liver segmentation performances (DSC, NSD, and HD95). **** represents *p* value < 0.0001. DL, deep learning; DSC, dice similarity coefficient; NSD, normalized surface dice.

In the independent test dataset of preoperative 44 CT scans with focal lesions, our DL algorithm statistically outperformed the manual method on the liver segmentation (DSC 0.98 ± 0.01 vs. 0.97 ± 0.01, NSD 0.89 ± 0.05 vs. 0.79 ± 0.05, HD95 1.88 ± 0.58 vs. 3.07 ± 2.03 for DL vs. manual method, respectively, all *p* < 0.001) (Figure 4 and Table 4). The comparisons between the DL and manual method on the liver segmentation were demonstrated in Figure 6.

**TABLE 4:** Quantitative comparison of segmentation performance between the DL and manual method.

		DSC			NSD			HD95		
Performance	Group	M ± SE	95%CI	*p*	M ± SE	95%CI	*p*	M ± SE	95%CI	*p*
Liver	DL	0.98 ± 0.01	(0.98, 0.98)	<0.001	0.89 ± 0.05	(0.87, 0.90)	<0.001	1.88 ± 0.58	(1.70, 2.05)	<0.001
	Manual	0.97 ± 0.01	(0.97, 0.97)		0.79 ± 0.05	(0.78, 0.80)		3.07 ± 2.03	(2.46, 3.69)	

		DSC			Recall			Precision		
Hepatic vessel	Group	M ± SE	95%CI	p	M ± SE	95%CI	p	M ± SE	95%CI	*p*
Hepatic veins	DL	0.86 ± 0.04	(0.85, 0.87)	<0.001	0.88 ± 0.05	(0.86, 0.89)	<0.001	0.84 ± 0.05	(0.83, 0.86)	<0.001
	Manual	0.52 ± 0.20	(0.46, 0.58)		0.59 ± 0.23	(0.52, 0.66)		0.57 ± 0.20	(0.51, 0.63)	
Portal veins	DL	0.90 ± 0.02	(0.90, 0.91)	<0.001	0.91 ± 0.03	(0.90, 0.92)	<0.001	0.90 ± 0.03	(0.89, 0.91)	<0.001
	Manual	0.58 ± 0.09	(0.56, 0.61)		0.62 ± 0.09	(0.60, 0.65)		0.58 ± 0.17	(0.53, 0.63)	
Inferior vena cava	DL	0.90 ± 0.08	(0.87, 0.92)	<0.001	0.87 ± 0.13	(0.83, 0.91)	<0.001	0.94 ± 0.03	(0.93, 0.95)	<0.001
	Manual	0.74 ± 0.08	(0.72, 0.76)		0.72 ± 0.13	(0.68, 0.75)		0.79 ± 0.09	(0.76, 0.82)	

*Note*: Data are represented as mean ± standard deviation while the data in parentheses are range. Comparison of DSC was calculated via the paired samples *t*‐test, while the NSD, HD95, Recall and Precision comparisons were computed using paired samples Wilcoxon signed rank test. *p* values < 0.001 show statistically significant differences.

Abbreviations: DL, deep learning; DSC, dice similarity coefficient; NSD, normalized surface dice; HD95, Hausdorff distance_95; M ± SE, mean ± standard; 95%CI, 95% confidence interval.

**FIGURE 5:**
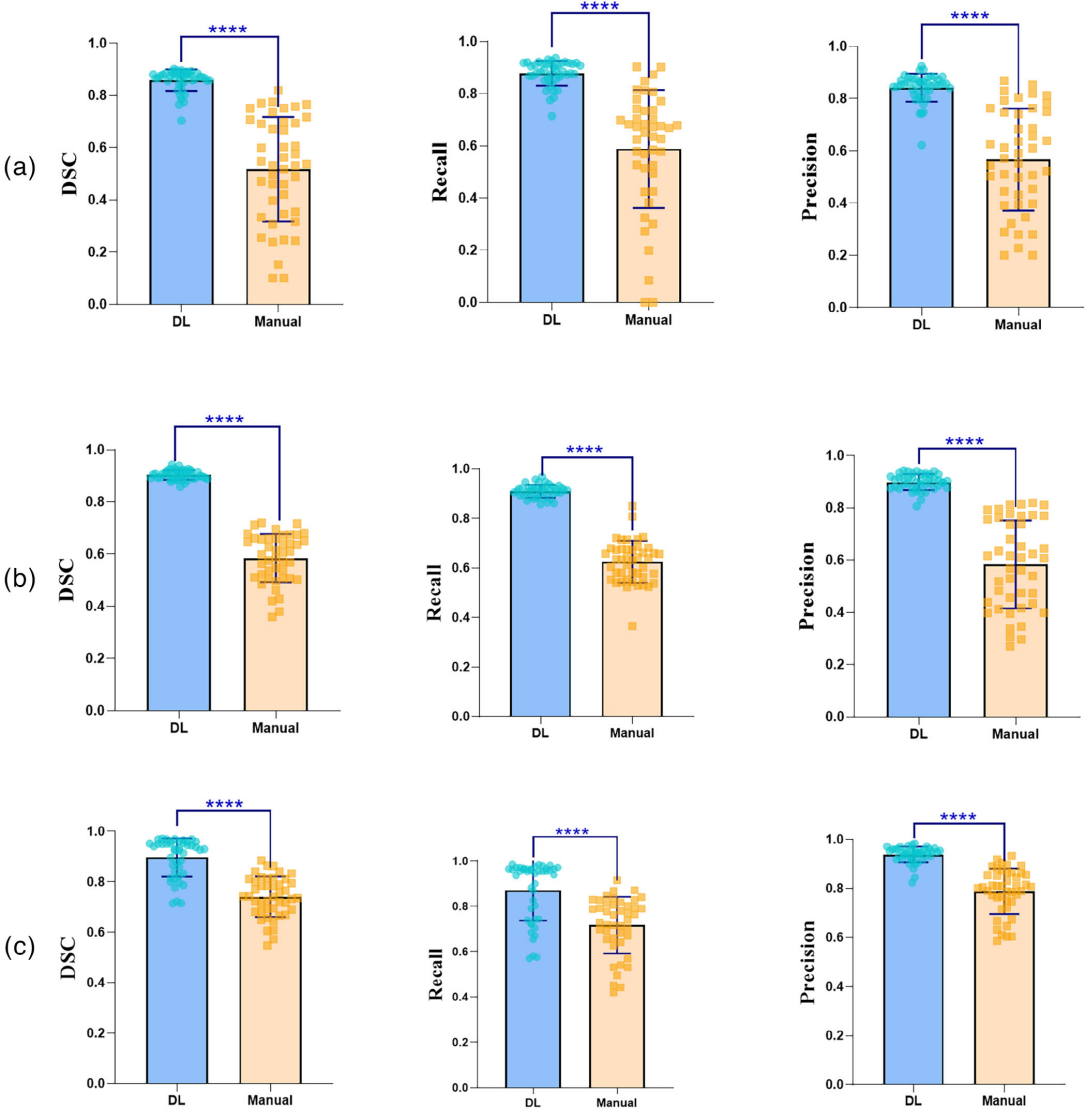
Box and scatter plots of the comparisons between the DL algorithm and manual method on the hepatic vessel segmentation performances (DSC, Recall, and Precision). **** represents *p* value < 0.0001. (a) Hepatic vein; (b) Portal vein; (c) Inferior vena cava. DL, deep learning; DSC, dice similarity coefficient.

**FIGURE 6:**
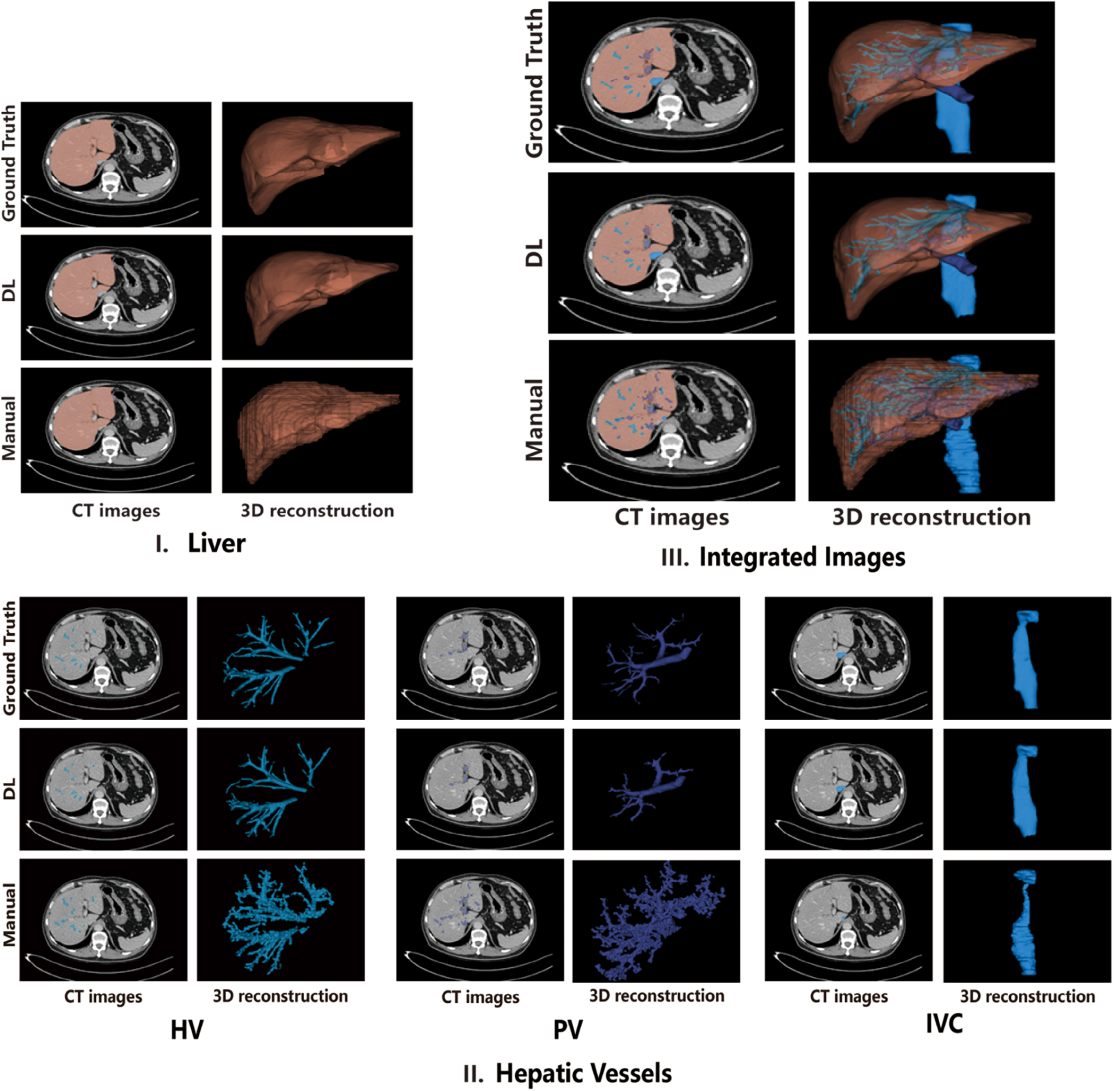
Comparisons between DL and manual method on the representative segmentation and 3D reconstruction results. Comparisons involve the segmentation performance of (I) Liver, (II) Hepatic vessels (hepatic vein, portal vein, and inferior vena cava), (III) Integrated of liver and hepatic vessels. In each imaging group, each row illustrates the segmentation results and 3D reconstruction based on the segmentation of the ground truth (first row), DL algorithm (second row), and manual approach (third row). DL, deep learning; HV, hepatic vein; IVC, inferior vena cava; PV, portal vein.

Similarly, the DL‐based segmentation quantitatively exceeded the manual approach for hepatic veins (DSC 0.86 ± 0.04 vs. 0.52 ± 0.20, Recall 0.88 ± 0.05 vs. 0.59 ± 0.23, Precision 0.84 ± 0.05 vs. 0.57 ± 0.20 for DL vs. manual method, respectively, all *p* < 0.001) (Figure 5a), portal veins (DSC 0.90 ± 0.02 vs. 0.58 ± 0.09, Recall 0.91 ± 0.03 vs. 0.62 ± 0.09, Precision 0.90 ± 0.03 vs. 0.58 ± 0.17 for DL vs. manual method, respectively, all *p* < 0.001) (Figure 5b) and inferior vena cava (DSC 0.90 ± 0.08 vs. 0.74 ± 0.08, Recall 0.87 ± 0.13 vs. 0.72 ± 0.13, Precision 0.94 ± 0.03 vs. 0.79 ± 0.09 for DL vs. manual method, respectively, all *p* < 0.001) (Figure 5c) (Table 4). The comparisons on the representative segmentation results were presented (Figure 6), as well as the integrated images of the liver and hepatic vessel segmentation. Additionally, the mean processing time of the DL algorithm was 173.2 s, which was significantly less than manual segmentation with 1032.7 s (p < 0.001) (Table 5).

**TABLE 5:** Comparison of the processing time between the DL and manual method.

Group	*N*	M ± SE	95%CI	*p*
DL	44	173.2 ± 68.5	(117.5, 201.0)	<0.001
Manual	44	1032.7 ± 242.7	(924.0, 1256.2)	

*Note*: Data are represented as mean ± standard deviation while the data in parentheses are range. M ± SE = mean ± standard; 95%CI = 95% confidence interval. Comparison of processing time was calculated via paired samples Wilcoxon signed rank test and *p* value < 0.001 show a statistically significant difference.

Compared to other networks, our proposed method showed superior performance in liver and hepatic vessel segmentation (Table 6).

**TABLE 6:** Comparisons between our proposed DL‐based and other networks.

	Liver			Hepatic vein			Portal vein			Inferior vena cava		
Networks	DSC	NSD	HD95	DSC	Recall	Precision	DSC	Recall	Precision	DSC	Recall	Precision
**CNN** **^16^**	0.83^*^	0.81^*^	3.78^*^	0.71^*^	0.75^*^	0.69^*^	0.76^*^	0.79*	0.71^*^	0.78^*^	0.79^*^	0.76^*^
**RA‐UNET** **^26^**	0.96^*^	0.89	1.95^*^	0.81^*^	0.79^*^	0.82^*^	0.85^*^	0.88^*^	0.83^*^	0.88^*^	0.89^*^	0.84^*^
**Vesselnet** **^27^**	0.92^*^	0.84^*^	2.37*	0.86	0.91^*^	0.73^*^	0.90	0.91	0.85^*^	0.90	0.93^*^	0.90^*^
**Our method**	0.98	0.89	1.88	0.86	0.88	0.84	0.90	0.91	0.90	0.90	0.87	0.94

Abbreviations: DSC, dice similarity coefficient; DL, deep learning; 95%CI, 95% confidence interval.

* Represents a significant difference when compared to our proposed DL‐based method using the paired samples *t*‐test.

### 3.3 Inter‐observer and inter‐method agreement

**FIGURE 7:**
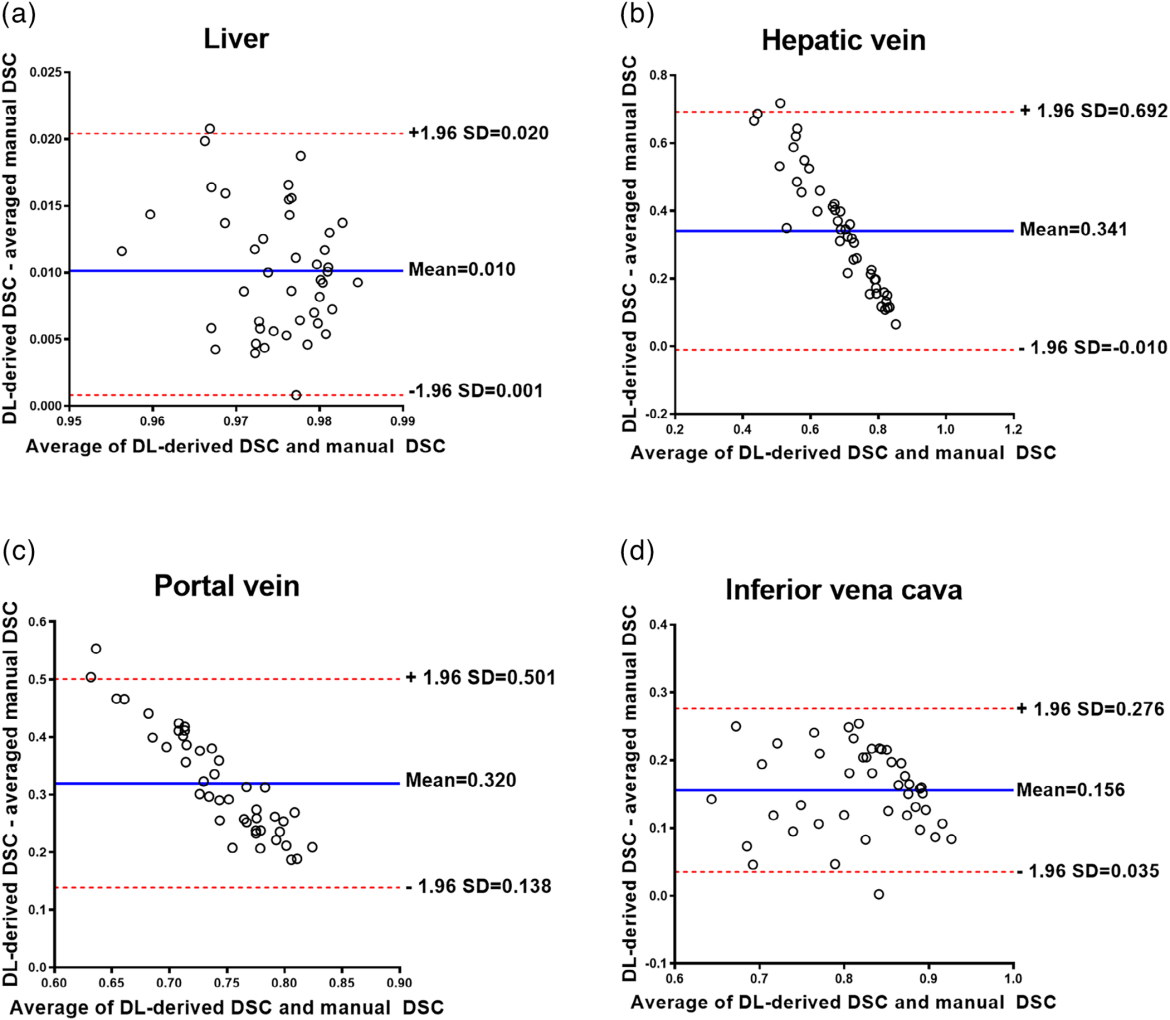
Bland–Altman plots for agreement between DSC values by the DL‐based and manual segmentations for liver (a), hepatic vein (b), portal vein (c), and inferior vena cava (d). Solid lines indicate mean differences and dashed lines indicate upper and lower limits of 95% limits of agreement. DSC, dice similarity coefficient; DL, deep learning; SD, standard deviation.

We found excellent ICCs above 0.992 for the inter‐reader assessment of the manual segmentations for liver and hepatic vessels (Table 7). In addition, there was an excellent agreement between the DL‐derived and manual‐derived results averaged over the three readers (Figure 7). The Bland–Altman 95% LOAs between the DL and manual approach were 0.010 ± 0.009 for the liver, 0.341 ± 0.351 for hepatic vein, 0.320 ± 0.181 for portal vein and 0.156 ± 0.121 for inferior vena cava (Table 7).

**TABLE 7:** Agreement of DSC values for the inter‐reader assessment and the inter‐method assessment.

Assessment	Liver	Hepatic vein	Portal vein	Inferior vena cava
**Inter‐reader Assessment**	**ICCs (95% CI)**			
Reader 1 versus 2	0.997 (0.986, 0.999)	0.997 (0.990, 0.999)	0.995 (0.989, 0.998)	0.998 (0.990, 0.999)
Reader 1 versus 3	0.994 (0.990, 0.996)	0.993 (0.985, 0.997)	0.994 (0.988, 0.996)	0.995 (0.986, 0.998)
Reader 2 versus 3	0.993 (0.988, 0.996)	0.992 (0.981, 0.996)	0.992 (0.982, 0.996)	0.996 (0.993, 0.998)
**Inter‐method Assessment**	**Bland–Altman 95% LOA (95% LOA)**			
DL‐derived versus manual method	0.010 ± 0.009 (0.001, 0.020)	0.341 ± 0.351 (−0.010, 0.692)	0.320 ± 0.181 (0.138, 0.501)	0.156 ± 0.121 (0.035, 0.276)

*Note*: The inter‐reader and inter‐method agreements were assessed using the intraclass correlation coefficient (ICCs) with corresponding 95 % confidence intervals (CI) and the Bland–Altman 95% limits of agreement (LOA), respectively. The Bland–Altman 95% LOA results are presented as the mean difference ± 1.96 × standard deviation (SD) of the difference and the limits of agreement are shown in parentheses.

Abbreviations: CI, confidence intervals; DSC, dice similarity coefficient; ICC, intraclass correlation coefficient; LOA, limits of agreement; SD, standard deviation.

## 4 DISCUSSION

In our study, we innovatively developed and validated a DL algorithm for automated liver and hepatic vessel segmentation using a large amount of annotated clinical CT data. Based on the original performance, our developed DL algorithm initially reached acceptable accuracy for both liver and hepatic vessel segmentation. In the independent comparison with the manual approach using preoperative images from clinical scenarios, DL‐based segmentation quantitively outperformed manual method with higher accuracy and processing efficiency, clinically indicating the robustness and generalizability of our DL algorithm. Compared to other CNN and Unet networks, our proposed method also showed superior performance in liver and hepatic vessel segmentation. The inter‐reader agreement assessments showed excellent results with regards to the ICC values. The DSC values obtained by DL‐based segmentation showed close agreement with those derived by the radiologists manually, with a small bias and measurement error.

Recently, several automated methods have been proposed for liver and hepatic vessel segmentation. A multi‐scale UNet proposed by Kushnure et al.^32^ using 20 CT scans from the 3Dircadb publicly available dataset achieved a DSC of 0.971 for liver segmentation and reduced the computational complexity. Wang et al.^33^ developed a multi‐scale attention and deep supervision‐based 3D UNet on three public datasets (including 131, 20 and 20 CT scans from LiTS17, SLiver07, and 3DIRCADb, respectively) with high accuracy (Dice of 0.9727, 0.9752, and 0.9691, respectively) for liver segmentation. Instead of using limited training data from public datasets with incomplete annotations, our DL algorithm was developed and evaluated using sufficient data with double‐refined annotations from our institution, generating results closer to clinical practice. Moreover, data augmentation was conducted to enrich the imaging appearances of liver and hepatic vessels to improve the robustness of the training and avoid data overfitting. Regardless of the higher DSC attained by our DL algorithm, NSD and HD95 were supplemented as additional metrics to comprehensively evaluate the DL‐based liver segmentation for surgical planning purposes. Since DSC sometimes cannot reflect the boundary errors of segmentation, NSD, as a more sensitive metric to the boundary errors, tends to quantitively assess the errors occurring between the boundaries of segmentation and ground truth.^34^ Especially in surgical planning such as needle trajectory planning, boundary errors are of vital importance and should be eliminated as little as possible.^35^, ^36^ The results of NSD and HD95 complementally indicated fewer boundary errors in the shape surface annotations and partly met the clinical demands for surgical planning. As for hepatic vascular segmentation, Kitrungrotsakul et al.^27^ applied deep convolutional networks with multi pathways to liver vessel segmentation and achieved the average DSC of 0.901 on the VASCUSYNTH simulation dataset and the highest Recall and Precision of 0.89 and 0.87 on the IRCAD dataset comprising 20 scans at 1% noise level, respectively. Hao et al.^37^ proposed a dual‐branch progressive 3D Unet for accurate segmentation of liver vessels and reached average DSC and sensitivity of 75.18% and 78.84% using public dataset 3Dircadb, respectively. However, these algorithms were trained and validated on limited training samples using incomplete annotations as the benchmark for training and evaluation, potentially causing huge bias. Different from the liver parenchymal, the proportions of hepatic vessels and background voxels are highly unbalanced and skewed. Due to the low contrast with surrounding tissues, high noise and irregular vessel shapes caused by nearby tumors, accurate liver vessel segmentation remains challenging.^38^ To improve the segmentation accuracy with imbalanced classes, the Tversky loss function was used to adjust the parameters of over‐ or under‐segmented foreground pixel numbers based on the dice loss function and increase the penalty for misclassified voxels to train and optimize the network to identify vessels with weak boundary, high noise or low contrast. Considering that our manual annotations were double‐checked and refined by experienced radiologists, inaccurate annotations were limited as far as possible in the datasets, leading to higher quality training and evaluating procedures. Based on the comparable accuracy to the previous results, the proposed DL algorithm effectively extracted liver vessel features from CI images and reconstructed the 3D position relationships of liver vessels. Inevitably, some tiny errors and discontinuities occurred, mainly in distal branches of hepatic veins, which minimally affected the surgical planning procedures.

To clinically confirm the adaptability to surgical planning scenarios, the accuracy and efficiency of DL‐based segmentation were independently evaluated and compared with manual approach using preoperative CT data from patients with intrahepatic focal lesions. By contrast, DL‐based segmentation achieved statistically higher accuracy with lower dispersion in data distribution, contributing to robust segmentation results without inter‐observer variability. Since various hepatic lesions within images did not significantly reduce the accuracy, the clinical generalizability was partly verified in CT images containing focal lesions, potentially allowing for larger‐scale application to patients with suspicious focal lesions. Although manual delineation and reconstruction possibly reached higher accuracy without the limitation of time and patience, similar accuracy was rarely reached in a clinically reasonable period, especially in surgical planning settings.^39^ In our study, the mean processing time of the DL algorithm was 173s, nearly six times faster than the 1032s of manual approach, representing higher efficiency in practice. Based on a coarse‐to‐fine pipeline, our algorithm rapidly smoothed the edged boundaries and surfaces of the segmentation leading to initial 3D spatial visualization in the coarse stage; the errors and discontinuities occurring at the vessel branches were further suppressed to ensure the integrity of vascular branches in the fine stage. To reduce computation and improve spatial connections, ConvMLP block was used to improve the network in a more lightweight and stage‐wise manner.

There are several limitations in our algorithm. Firstly, it was developed and evaluated using single‐center data. Though we utilized sufficient numbers of data with high‐quality annotations, the broad applicability should be further assessed based on external data from multiple institutions. Secondly, our algorithm only offers the segmentation of the liver and vessels, but not hepatic lesions or segmental liver. DL‐based segmentation aimed at the hepatic focal lesions and functional segments has already been planned in our further research. Thirdly, only the portal venous phase CT images were used in the algorithm training, potentially limiting the applications. The larger‐scale applications to other imaging modalities require further training based on additional training data through transfer learning. Fourthly, our algorithm was not systematically compared with state‐of‐the‐art networks. Lastly, a small portion of errors and discontinuities still exist in some vessel segmentation with lower accuracy, which undoubtedly needs to be solved by optimizing our model.

## 5 CONCLUSION

In conclusion, we developed and evaluated a DL algorithm for automated liver and hepatic vessel segmentation based on large amounts of CT images. Compared with the manual approach, our DL algorithm quantitatively showed higher accuracy with less time consumption. Our algorithm potentially serves as a CT‐based practical tool to clinically assist surgical planning.

## AUTHOR CONTRIBUTIONS

Xiaoguang Li led and coordinated this study. Shengwei Li and Lin Cheng collected the data and built the datasets. Fanyu Zhou, Yumeng Zhou, and Shengwei Li trained and developed the deep learning algorithm. Zhixin Bie led and performed the manual segmentation work with Jingzhao Peng and Bin Li. Shengwei Li performed data interpretation and statistical analysis under the supervision of Xiaoguang Li. Shengwei Li was the major contributor to writing and revising the manuscript. All authors were involved in critical revisions of the manuscript, and have read and approved the final version.

## CONFLICT OF INTEREST STATEMENT

The authors have no relevant financial or non‐financial interests to disclose.

## ETHICS STATEMENT

This study was approved by the Institutional Review Board of Beijing Hospital (IRB No. 2022‐BJYYEC‐361‐01). This study was performed in line with the principles of the Declaration of Helsinki. Written informed consent was waived due to the retrospective nature of our study.

## Supporting information

Supporting information

Supporting information

## Data Availability

The imaging data from Beijing Hospital currently cannot be publicly accessible due to privacy protection. Reasonable requests for the datasets and materials used in this study can be addressed to the corresponding author.

## References

[1] Gotra A , Sivakumaran L , Chartrand G , et al. Liver segmentation: indications, techniques and future directions. Insights Imaging. 2017;8(4):377‐392. 28616760 10.1007/s13244-017-0558-1 PMC5519497

[2] Ahn Y , Yoon JS , Lee SS , et al. Deep learning algorithm for automated segmentation and volume measurement of the liver and spleen using portal venous phase computed tomography images. Korean J Radiol. 2020;21(8):987‐997. 32677383 10.3348/kjr.2020.0237 PMC7369202

[3] Nayantara PV , Kamath S , Manjunath KN , Rajagopal KV . Computer‐aided diagnosis of liver lesions using CT images: a systematic review. Comput Biol Med. 2020;127:104035. 33099219 10.1016/j.compbiomed.2020.104035

[4] Alirr OI , Rahni AAA . Hepatic vessels segmentation using deep learning and preprocessing enhancement. J Appl Clin Med Phys. 2023;24(5):e13966. 36933239 10.1002/acm2.13966 PMC10161019

[5] Tian Y , Xue F , Lambo R , et al. Fully‐automated functional region annotation of liver via a 2.5D class‐aware deep neural network with spatial adaptation. Comput Methods Programs Biomed. 2021;200:105818. 33218708 10.1016/j.cmpb.2020.105818

[6] Anderson BM , Rigaud B , Lin YM , et al. Automated segmentation of colorectal liver metastasis and liver ablation on contrast‐enhanced CT images. Front Oncol. 2022;12:886517. 36033508 10.3389/fonc.2022.886517 PMC9403767

[7] Shi C , Xian M , Zhou X , Wang H , Cheng HD . Multi‐slice low‐rank tensor decomposition based multi‐atlas segmentation: application to automatic pathological liver CT segmentation. Med Image Anal. 2021;73:102152. 34280669 10.1016/j.media.2021.102152

[8] Lebre MA , Vacavant A , Grand‐Brochier M , et al. Automatic segmentation methods for liver and hepatic vessels from CT and MRI volumes, applied to the Couinaud scheme. Comput Biol Med. 2019;110:42‐51. 31121506 10.1016/j.compbiomed.2019.04.014

[9] Yang X , Yang JD , Hwang HP , et al. Segmentation of liver and vessels from CT images and classification of liver segments for preoperative liver surgical planning in living donor liver transplantation. Comput Methods Programs Biomed. 2018;158:41‐52. 29544789 10.1016/j.cmpb.2017.12.008

[10] Liu Y , Wang Q , Du B , Wang X , Xue Q , Gao W . A meta‐analysis of the three‐dimensional reconstruction visualization technology for hepatectomy. Asian J Surg. 2023;46(2):669‐676. 35843827 10.1016/j.asjsur.2022.07.006

[11] Yu H , Sharifai N , Jiang K , et al. Artificial intelligence based liver portal tract region identification and quantification with transplant biopsy whole‐slide images. Comput Biol Med. 2022;150:106089. 36137315 10.1016/j.compbiomed.2022.106089

[12] Chen Y , Zheng C , Hu F , et al. Efficient two‐step liver and tumour segmentation on abdominal CT via deep learning and a conditional random field. Comput Biol Med. 2022;150:106076. 36137320 10.1016/j.compbiomed.2022.106076

[13] Lebre MA , Vacavant A , Grand‐Brochier M , et al. A robust multi‐variability model based liver segmentation algorithm for CT‐scan and MRI modalities. Comput Med Imaging Graph. 2019;76:101635. 31301489 10.1016/j.compmedimag.2019.05.003

[14] Li R , Huang YJ , Chen H , et al. 3D graph‐connectivity constrained network for hepatic vessel segmentation. IEEE J Biomed Health Inform. 2022;26(3):1251‐1262. 34613925 10.1109/JBHI.2021.3118104

[15] Guo X , Xiao R , Zhang T , Chen C , Wang J , Wang Z . A novel method to model hepatic vascular network using vessel segmentation, thinning, and completion. Med Biol Eng Comput. 2020;58(4):709‐724. 31955327 10.1007/s11517-020-02128-6

[16] Huo Y , Terry JG , Wang J , et al. Fully automatic liver attenuation estimation combing CNN segmentation and morphological operations. Med Phys. 2019;46(8):3508‐3519. 31228267 10.1002/mp.13675 PMC6692233

[17] Liu M , Vanguri R , Mutasa S , Ha R , Liu YC , Button T , et al. Channel width optimized neural networks for liver and vessel segmentation in liver iron quantification. Comput Biol Med. 2020;122:103798. 32658724 10.1016/j.compbiomed.2020.103798

[18] Balasubramanian PK , Lai WC , Seng GH , Ccc K , Selvaraj J . APESTNet with mask R‐CNN for liver tumor segmentation and classification. Cancers (Basel). 2023;15(2):330. 36672281 10.3390/cancers15020330 PMC9857237

[19] Luu MH , Mai HS , Pham XL , et al. Quantification of liver‐Lung shunt fraction on 3D SPECT/CT images for selective internal radiation therapy of liver cancer using CNN‐based segmentations and non‐rigid registration. Comput Methods Programs Biomed. 2023;233:107453. 36921463 10.1016/j.cmpb.2023.107453

[20] Shelhamer E , Long J , Darrell T . Fully convolutional networks for semantic segmentation. IEEE Trans Pattern Anal Mach Intell. 2017;39(4):640‐651. 27244717 10.1109/TPAMI.2016.2572683

[21] Chen LC , Papandreou G , Kokkinos I , Murphy K , Yuille AL . DeepLab: semantic image segmentation with deep convolutional nets, atrous convolution, and fully connected CRFs. IEEE Trans Pattern Anal Mach Intell. 2018;40(4):834‐848. 28463186 10.1109/TPAMI.2017.2699184

[22] Huang G , Liu Z , Van Der Maaten L , Weinberger KQ , editors. Densely connected convolutional networks. Proceedings of the IEEE conference on computer vision and pattern recognition; 2017.

[23] He K , Zhang X , Ren S , Sun J , editors. Deep residual learning for image recognition. Proceedings of the IEEE conference on computer vision and pattern recognition; 2016.

[24] Goodfellow I , Pouget‐Abadie J , Mirza M , et al. Generative adversarial networks. 2020;63(11):139‐144.

[25] Ronneberger O , Fischer P , Brox T . U‐Net: convolutional networks for biomedical image segmentation. Medical Image Computing and Computer‐Assisted Intervention—MICCAI 2015. Springer International Publishing; 2015. 2015//.

[26] Wang J , Zhang X , Lv P , Wang H , Cheng Y . Automatic liver segmentation using EfficientNet and attention‐based residual U‐Net in CT. J Digit Imaging. 2022;35(6):1479‐1493. 35711074 10.1007/s10278-022-00668-x PMC9712863

[27] Kitrungrotsakul T , Han XH , Iwamoto Y , et al. VesselNet: a deep convolutional neural network with multi pathways for robust hepatic vessel segmentation. Comput Med Imaging Graph. 2019;75:74‐83. 31220699 10.1016/j.compmedimag.2019.05.002

[28] Gul S , Khan MS , Bibi A , Khandakar A , Ayari MA , Chowdhury MEH . Deep learning techniques for liver and liver tumor segmentation: a review. Comput Biol Med. 2022;147:105620. 35667155 10.1016/j.compbiomed.2022.105620

[29] Li Y , Zhu Z , Zhou Y , et al., editors. Volumetric Medical Image Segmentation: A 3D Deep Coarse‐to‐Fine Framework and Its Adversarial Examples. Deep Learning and Convolutional Neural Networks for Medical Imaging and Clinical Informatics; 2020.

[30] Li J , Hassani A , Walton S , Shi HJA , ConvMLP: Hierarchical Convolutional MLPs for Vision; 2021;abs/2109.04454.

[31] Moccia S , De Momi E , El Hadji S , Mattos LS . Blood vessel segmentation algorithms—Review of methods, datasets and evaluation metrics. Comput Methods Programs Biomed. 2018;158:71‐91. 29544791 10.1016/j.cmpb.2018.02.001

[32] Kushnure DT , TalbarSN . MS‐UNet: a multi‐scale UNet with feature recalibration approach for automatic liver and tumor segmentation in CT images. Comput Med Imaging Graph. 2021;89:101885. 33684731 10.1016/j.compmedimag.2021.101885

[33] Wang J , Zhang X , Guo L , Shi C , Tamura S . Multi‐scale attention and deep supervision‐based 3D UNet for automatic liver segmentation from CT. Math Biosci Eng. 2023;20(1):1297‐1316. 36650812 10.3934/mbe.2023059

[34] Ma J , Zhang Y , Gu S , et al. AbdomenCT‐1K: is abdominal organ segmentation a solved problem? IEEE Trans Pattern Anal Mach Intell. 2022;44(10):6695‐6714. 34314356 10.1109/TPAMI.2021.3100536

[35] Ni ZK , Lin D , Wang ZQ , et al. Precision liver resection: three‐dimensional reconstruction combined with fluorescence laparoscopic imaging. Surg Innov. 2021;28(1):71‐78. 32873180 10.1177/1553350620954581

[36] Tong N , Xu Y , Zhang J , Gou S , Li M . Robust and efficient abdominal CT segmentation using shape constrained multi‐scale attention network. Phys Med. 2023;110:102595. 37178624 10.1016/j.ejmp.2023.102595

[37] Hao W , Zhang J , Su J , et al. HPM‐Net: hierarchical progressive multiscale network for liver vessel segmentation in CT images. Comput Methods Programs Biomed. 2022;224:107003. 35868034 10.1016/j.cmpb.2022.107003

[38] Huang Q , Sun J , Ding H , Wang X , Wang G . Robust liver vessel extraction using 3D U‐Net with variant dice loss function. Comput Biol Med. 2018;101:153‐162. 30144657 10.1016/j.compbiomed.2018.08.018

[39] Koitka S , Gudlin P , Theysohn JM , et al. Fully automated preoperative liver volumetry incorporating the anatomical location of the central hepatic vein. Sci Rep. 2022;12(1):16479. 36183002 10.1038/s41598-022-20778-4 PMC9526715

