# Supplementary material for: Automated segmentation of liver and hepatic vessels on portal venous phase computed tomography images using a deep learning algorithm
Source: J Appl Clin Med Phys. 2024 May 21;25(8):e14397. doi: 10.1002/acm2.14397 (PMC11302809; doi:10.1002/acm2.14397)
Supplement: Supplementary file 1 — Supporting information [file ACM2-25-e14397-s002.docx]

**The proposed DL algorithm**

1. **DL algorithm architecture**

As to the encoder module, the consecutive multi-layer perceptron (ConvMLP) block consists of Channelwise multi-layer perceptrons (MLPs), with skip layers, and a 3×3×3 depth-wise convolution between the two channel MLPs in each MLP block. Despite the additional computational time and load to the network, the utilization of MLP allows for improved accuracy. This is repeated 4 times before a down convolution is utilized. The structure of a ConvMLP block usually consists of a series of fully connected layers (MLP), where each layer takes each position in the input feature map as input and produces a new feature vector. These feature vectors undergo position-independent operations to capture global and local feature information. By stacking multiple ConvMLP blocks, increasingly higher-level feature representations can be extracted.

As to the decoder module, the residual convolution block is implemented as convolution-InstanceNorm-ReLU (conv-instnorm-ReLU), where the addition of the residual takes place before the last ReLU activation. We adopt a 3D-based mixed pyramid pooling to extract contextual features, which is composed of standard spatial pooling^[31]^. The standard spatial pooling employs two average pooling with the stride of 2×2×2 and 4×4×4. The initial number of feature maps is 8 for the coarse model and 16 for the fine model.

**2. Loss function**

Our image-segmentation DL framework used the Tversky loss function, a loss function calculated from the Dice loss (L_Dice_) and CE loss (cross-entropy loss, L_CE_) to comprehensively balance the recall and precision of segmentation results. In the equation below, α is a parameter to weigh the Dice and CE loss and is set to 0.5 in this study to equally balance the recall and precision.

$$L=\alpha\times L_{Dice}+(1-\alpha)\times L_{CE}$$

To mitigate poor segmentation results for vessels caused by the class imbalance in dataset, we modified the loss function and introduced an adapted softmax loss function with adaptive weights. The weighted loss function is computed as follows:

$$l\left( y,z \right)= -\sum_{k=0}^{c} w_{c}y_{c}log(f(z_{c}))$$

In the equation above, c is the number of classes in the neural network. $w_{c}$is the weight for class c. $y_{c}$represents the actual label value. $z_{c}$is the input to the softmax function. $f(z_{c})$represents the output of the softmax function, indicating the probability of the sample belonging to class c.

To calculate the contribution of each loss to the total loss, we allocate weights to each loss based on their proportions. Each loss is multiplied by the new weight, and then all losses are summed to obtain the reweighted total loss.

**3. Training process and implementation**

Our DL algorithm was trained and validated using 413 and 52 CT scans, respectively. The training protocols are shown in Supplementary Table 1.

The DL algorithm was implemented using Pytorch 1.12 in Python 3.8. The models were trained computers with AMD Ryzen Threadripper 3990X 64-Core Processor CPU with 256 GB RAM, and four NVIDIA GeForce RTX 3090 GPU (4x24GB memory). Considering the GPU memory limitation, the batch size of the coarse and fine models was set to 2 and 4, respectively.

Supplementary Table 1. Training protocols of the proposed DL algorithm

| Initialization of the network | Kaiming normal initialization |
| --- | --- |
| Patch sampling strategy | Augment the sample ratio of the pathological image (3 times) |
| Batch size | Coarse: 4  Fine: 2 |
| Patch size | Coarse: 128*×*128*×*128  Fine: 256*×*256*×*256 |
| Total epochs | 200 |
| Optimizer | Adam with betas (0.9, 0.99), L2 penalty: 0.00001 |
| Loss | Dice loss and focal loss (alpha = 0.5, gamma = 2) |
| Dropout rate | 0.2 |
| Initial learning rate | 0.01 |
| Learning rate decay schedule | Step decay |
| Stopping criteria, and optimal model selection criteria | The stopping criterion is reaching the maximum number of epochs (200). |
| Training mode | Mixed precision |
| Training time for coarse model | 3 hours |
| Training time for fine model | 6 hours |
