# Supplementary material for: Automated segmentation of liver and hepatic vessels on portal venous phase computed tomography images using a deep learning algorithm
Source: J Appl Clin Med Phys. 2024 May 21;25(8):e14397. doi: 10.1002/acm2.14397 (PMC11302809; doi:10.1002/acm2.14397)
Supplement: Supplementary file 2 — Supporting information [file ACM2-25-e14397-s001.docx]

**Definition of evaluation metrics**

Dice Similarity Coefficient (DSC) was the main evaluation metric to measure the region overlap error in a range from 0 to 1 (perfect segmentation), and a higher value indicates a better segmentation performance. The DSC definition is shown in the following:

$$DSC\left( G,S \right)= \frac{2\left| G\cap S \right|}{\left| G \right|+\left| S \right|}$$

where G and S denote the set of ground-truth delineation and segmentation results, respectively.

Normalized surface Dice (NSD) is a boundary-based measure to evaluate similarity between segmentation and ground truth surfaces at a specified tolerance τ, ranging 0 to 1, and a higher value represents better segmentation. The NSD definition is shown in the following:

$$NSD\left( G,S \right)=\frac{\left| \partial G\cap\partial B_{\partial S}^{\left( \tau\right)} \right|+\left| \partial S\cap\partial B_{\partial G}^{\left( \tau\right)} \right|}{\left| \partial G \right|+ \left| \partial S \right|}$$

$$B_{\partial G}^{\left( \tau\right)}=\{x \in R3| \exists x˜ \in\partial G, ||x - x˜|| \leq\tau\}$$

$$B_{\partial S}^{\left( \tau\right)}== \{x \in R3| \exists x˜ \in\partial S, ||x-x˜|| \leq\tau\}$$

where $\left| \partial G \right|$ and $\left| \partial S \right|$ represent the number of voxels of the ground truth and the segmentation results, respectively; where $B_{\partial G}^{\left( \tau\right)},B_{\partial S}^{\left( \tau\right)}$denote the border region of the ground truth and the segmentation surface at tolerance τ, respectively. In our study, the tolerance is set τ as 1mm.
Hausdorff distance_95 (HD95) is the 95th percentile of HD values, presents the 95% maximum distance from segmentation surface to the reference surface, and a lower value implies better segmentation. The definition of HD is shown as follows:

$$d_{H}\left\{ X,Y \right\}=max\left\{ d_{XY},d_{YX} \right\}=max\left\{ \max_{x\in X} \min_{y\in Y} d\left( x,y \right), \max_{y\in Y} \min_{x\in X} d\left( x,y \right) \right\}$$

where X and Y denote the boundary-surface set of the segmentation results and the ground truth, d (x, y) indicates the Euclidean distance between voxels x and y.

These two metrics are defined as follows:

$$Recall= \frac{TP}{TP+FN}$$

$$Precision= \frac{TP}{TP+FP}$$

where the TP, FP and FN denotes the true-positive, false-positive and false-negative cases.
